# Neurofeedback of Alpha Activity on Memory in Healthy Participants: A Systematic Review and Meta-Analysis

**DOI:** 10.3389/fnhum.2020.562360

**Published:** 2021-01-05

**Authors:** Wen-Hsiu Yeh, Jen-Jui Hsueh, Fu-Zen Shaw

**Affiliations:** ^1^Institute of Basic Medical Science, National Cheng Kung University, Tainan, Taiwan; ^2^Mind Research and Imaging Center, National Cheng Kung University, Tainan, Taiwan; ^3^Department of Psychology, National Cheng Kung University, Tainan, Taiwan

**Keywords:** alpha, cognition, memory, neurofeedback, randomized controlled trial

## Abstract

**Background:** Neurofeedback training (NFT) has recently been proposed as a valuable technique for cognitive enhancement and psychiatric amelioration. However, effect of NFT of alpha activity on memory is controversial. The current study analyzed previous works in terms of randomized and blinded analyses, training paradigms, and participant characteristics to validate the efficacy of alpha NFT on memory in a healthy population.

**Objectives:** A systematic meta-analysis of studies with randomized controlled trials was performed to explore the effect of alpha NFT on working memory (WM) and episodic memory (EM) in a healthy population.

**Methods:** We searched PubMed, Embase, and Cochrane Library from January 1, 1999, to November 30, 2019. Previous studies were evaluated with the Cochrane risk of bias (RoB). A meta-analysis calculating absolute weighted standardized mean difference (SMD) using random-effects models was employed. Heterogeneity was estimated using *I*^2^ statistics. Funnel plots and Egger's test were performed to evaluate the quality of evidence.

**Results:** Sixteen studies with 217 healthy participants in the control group and 210 participants in the alpha group met the eligibility criteria. Alpha NFT studies with WM measures presented little publication bias (*P* = 0.116), and 5 of 7 domains in the Cochrane RoB exhibited a low risk of bias. The overall effect size from 14 WM studies was 0.56 (95% CI 0.31–0.81, *P* < 0.0001; *I*^2^ = 28%). Six EM studies exhibited an effect size of 0.77 (95% CI 0.06–1.49, *P* = 0.03; *I*^2^ = 77%).

**Conclusion:** Meta-analysis results suggest that alpha NFT seems to have a positive effect on the WM and EM of healthy participants. Future efforts should focus on the neurophysiological mechanisms of alpha NFT in memory.

## Introduction

Electroencephalogram (EEG) consists of various brain activities, such as alpha, theta, or beta rhythm. Distinct brain activity reflects particular cognitive functions. For example, alpha activity is accompanied by a resting eye-closed state, which is related to relaxation and the cortical inhibition of the sensory cortex (Klimesch et al., 2007). In contrast, alpha activities of the frontal and parietal cortices have a highly positive correlation with intelligence in healthy adults, particularly memory (Klimesch, 1999; Doppelmayr et al., 2002). Pre-stimulus alpha activity also plays a role in attention and memory processing (Wang and Hsieh, 2013). Moreover, event-related synchronization or desynchronization within 8–12 Hz exhibits a high correlation with accurate motor performance (Ros et al., 2014). These findings suggest that alpha activity plays a specific role in cognitive modulation (Palva and Palva, 2007). It is of interest to investigate whether actively controlling alpha activity produces a positive cognitive effect.

EEG neurofeedback is an operant conditioning technique to achieve self-control of specific types of brain activity (Heinrich et al., 2007). The participant's control over his or her EEG activity is typically mediated with visual (Hsueh et al., 2016), auditory (Alekseeva et al., 2012), or combined feedback (Guez et al., 2014). EEG neurofeedback training (NFT) is a non-pharmacological approach and has been increasingly considered promising psychological training since the 1990s (Gruzelier, 2014). Currently, NFT is considered as a technique to improve cognitive function in healthy subjects or neurological/psychiatric patients (Luijmes et al., 2016; Steingrimsson et al., 2020). Previous review articles have indicated controversy regarding available NFTs for the amelioration of symptoms and/or improvement of cognitive function (including memory) in particular populations, such as patients with stroke (Renton et al., 2017), posttraumatic stress disorder (Steingrimsson et al., 2020), or attention-deficit/hyperactivity disorder (Cortese et al., 2016). Heterogeneity of neurological or psychiatric disorders accompanied by the alteration of different neural networks is always present in these patient populations and may present difficulty in determining conclusive effects of NFT. To ascertain the efficacy of NFT on memory, we targeted studies of healthy participants exclusively for meta-analysis to reduce substantial heterogeneity in the selected population.

The efficacy of alpha NFT on memory varies across studies. NFT of alpha activity exhibits significant enhancement of working memory (WM) (Zoefel et al., 2011; Nan et al., 2012) and/or episodic memory (EM) (Hsueh et al., 2016; Wei et al., 2017). Some articles have found little memory improvement throughout alpha NFT (Bauer, 1976; Boynton, 2001; Angelakis et al., 2007). These controversial results may arise from a weak experimental design [e.g., no control arm (Hanslmayr et al., 2005) non-random allocation (Bauer, 1976)], or little power due to a small population in previous studies. The available NFT studies present various training paradigms, including different numbers of training sessions and training duration of a session. All of these factors contribute to divergent results of alpha NFT on memory. It is necessary to perform a systematic review of the NFT of alpha activity on memory. A meta-analysis of available works may provide a good opportunity to elucidate the possible effect of alpha NFT on memory.

The present study aimed to explore alpha NFT on both WM and EM through a meta-analysis of available previous works in a healthy population. We summarized all previous works in terms of study bias (selection bias, detection bias, performance bias, etc.), training paradigm (electrode placement, feedback modality, training frequency, etc.), and participant characteristic (age and amount). Our work provides quantitative and qualitative information to evaluate whether alpha NFT is a viable intervention for memory in a healthy population.

## Methods

We followed the Preferred Reporting Items for Systematic Reviews and Meta-Analysis (PRISMA) recommendations to undertake the search and analysis of the international scientific literature (Moher et al., 2009, 2015).

### Data Sources and Searches

Literature searches were conducted in the following electronic bibliographic databases: PubMed, Embase and The Cochrane Library (Cochrane Central Register of Controlled Trials). The searches were conducted from 1st January 1999 to 30th November 2019. The search string was structured using the PICOS method: P (population) = None, I (intervention) = alpha neurofeedback, C (comparison) = no intervention, sham, or control group, O (outcome) = WM, EM, and cognition, and S (study design) = randomized controlled trial (RCT).

The following search terms were used in 3 electronic bibliographic databases: (Alpha OR alpha) AND (neurofeedback OR Neurofeedback OR Electroencephalographic biofeedback OR electroencephalographic biofeedback OR EEG biofeedback OR EEG Biofeedback) AND (memor^*^ OR Memor^*^ OR cogniti^*^ OR Cogniti^*^) AND (random group OR sham control OR sham group OR sham OR control OR control group OR non-alpha OR non-alpha group OR non-alpha control).

### Study Selection

We combined search results from different databases using EndNote reference manager software and deleted duplicate records. Then, two authors (WHY, JJH) independently screened the titles and abstracts to remove ineligible studies. One author (WHY) further evaluated the eligibility of these full-text articles. In case of doubt, the results were discussed among all authors.

Studies were included if they met the following criteria:

1. Design: RCT.
2. Intervention: standard protocol EEG-NFT of alpha activity, e.g., alpha peak amplitude, entire alpha amplitude, upper-band amplitude of an individual alpha frequency, theta/alpha ratio, or alpha and theta activities.
3. Control group: receiving active neurofeedback [e.g., randomly selected 4-Hz amplitude from the range of 7–20 (Hsueh et al., 2016) or 4–45 Hz (Pei et al., 2018)], sham neurofeedback [e.g., simulated EEG activity from others (Xiong et al., 2014)], or silent feedback [including non-neurofeedback (Gordon et al., 2019)].
4. Participants: healthy population.
5. Evaluation: alpha effect on WM and/or EM.

Studies were ineligible if they were not written in English or were conference abstracts.

### Study Bias Assessment

Study quality was assessed by the author WHY using the Cochrane risk of bias (RoB) tool (Higgins and Green, 2011). To ascertain the RoB of the eligible articles, the author determined the quality of each study with regard to selection bias, detection bias, performance bias, attrition bias, and reporting bias. Three levels, i.e., low, unclear, and high risk of bias, were used for evaluating each parameter.

### Data Extraction and Statistical Analysis

Data were extracted by WHY using a standardized data extraction form. For all included studies, information was gathered on the experimental design, population, EEG-alpha NFT characteristics (electrode positions, NFT type, number of sessions, and duration of a session), and results.

Outcomes of interest were alpha NFT on WM (e.g., backward digital span or mental rotation tasks) and EM (e.g., word pair task) in healthy participants. Data were extracted from the control and alpha NFT groups. We calculated the standardized mean difference (SMD) with 95% confidence intervals (CIs) of WM and EM in two groups for each study. To allow for variability among the participants and interventions, random effects modeling for pooled effect size (ES) was used because it provided a more conservative ES estimate (DerSimonian and Laird, 1986). The *I*^2^ statistic was used to quantify heterogeneity across studies, with values of 25, 50, and 75% reflecting a small, medium, or high degree of heterogeneity, respectively (Higgins et al., 2003). Statistically significant heterogeneity was present at *p* < 0.1. A forest plot was generated to show the SMD with the corresponding CIs for each study and the overall estimate of pooled random effects. Publication bias was assessed with funnel plots and Egger tests. Analyses were performed using Review Manager 5.3 software (Cochrane Collaboration, Copenhagen, Denmark) and STATA 15 (Higgins and Green, 2011). *P*-values for all comparisons were two-tailed, and *p* < 0.05 of all tests was considered statistically significant, except for heterogeneity.

## Results

### Study Selection

**Figure 1:**
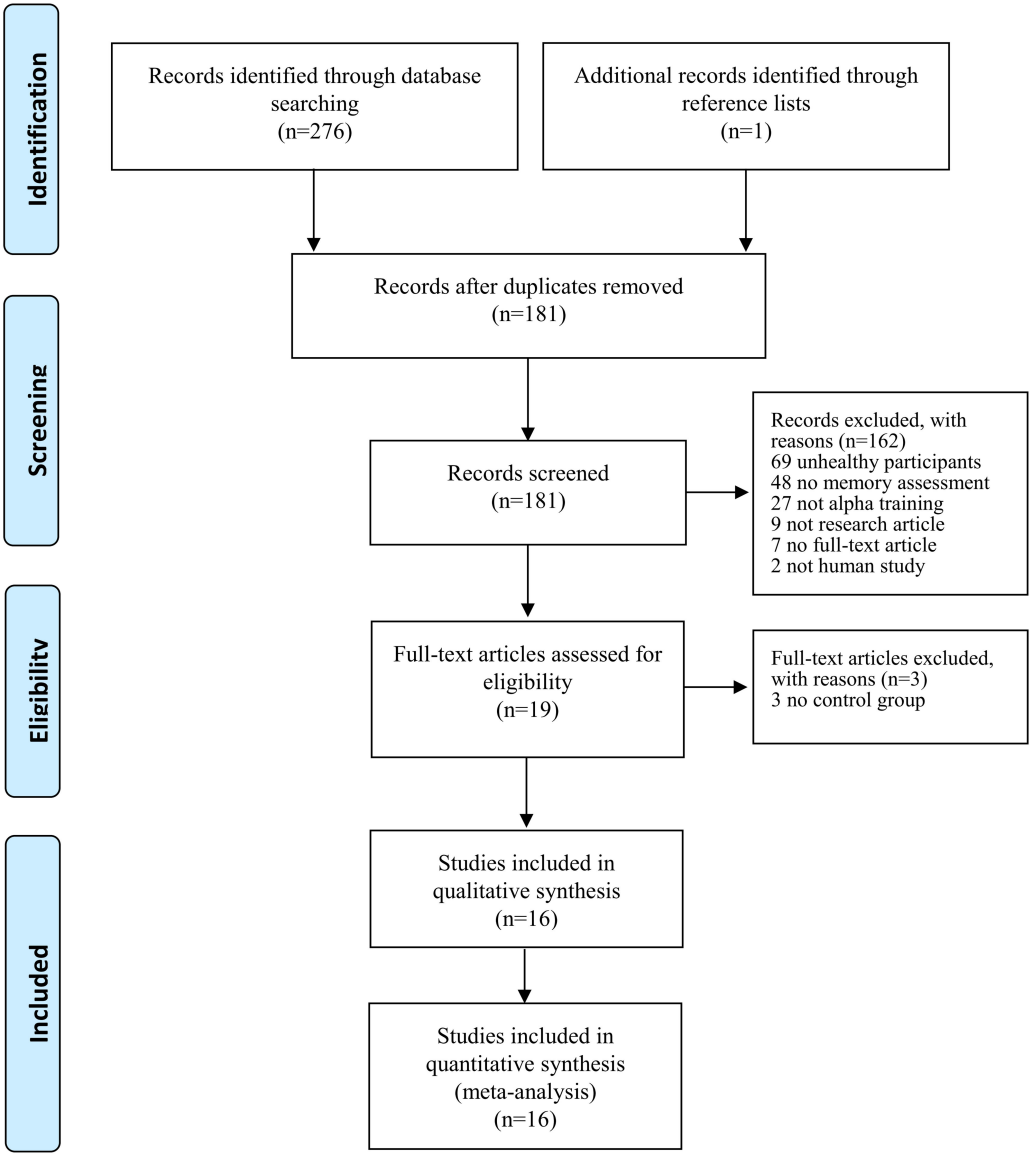
The Preferred Reporting Items for Systematic Reviews and Meta-Analyses (PRISMA) flow diagram presenting the literature searches and the included studies.

Figure 1 shows the schematic flow diagram for the process of study selection. A total of 277 titles and abstracts were initially identified through database searching (*n* = 276) and by checking relevant articles in reference lists (*n* = 1). After removal of 96 duplicates, 181 titles and abstracts were screened for relevance. One hundred and sixty-two studies did not meet the inclusion criteria: studies including patients (*n* = 69), absence of memory assessment (*n* = 48), no NFT of alpha activity (*n* = 27), not an original research article (*n* = 9), not a full-text article (e.g., poster or abstract) (*n* = 7), and not a human study (*n* = 2). Subsequently, 3 studies were excluded due to the lack of a sham or control group.

### Study Characteristics

A total of 16 papers met the criteria for the qualitative synthesis (Table 1). The studies with a RCT were published from 2011 to 2019. Among the included studies, 10 studies were two-arm RCTs, 3 studies were three-arm RCTs, 2 studies were four-arm RCTs, and 1 study was a six-arm RCT. Overall, the studies included healthy participants, with an accumulated population of 427 [ranging from 16 (Escolano et al., 2011) to 60 (Gordon et al., 2019) participants]. The mean age of the overall population was 28 years old.

**Table 1:** Characteristics of studies included in Meta-Analysis.

**References**	**Design**	**Sample**		**EEG-alpha NFT characteristics**			**Outcomes of interest**	
		**Participants**	**Age (mean ± SD)**	**Electrode(s)**	**Modality**	**Sessions (min/session)**	**WM**	**EM**
Alekseeva et al., 2012	Two-arm trial (Control vs. α)	27 (13 vs. 14)	(19.8 ± 0.6 vs. 19.5 ± 0.4)	Pz	Upper α	8 (18)	MRT	
Escolano et al., 2011	Two-arm trial (Control vs. α)	16 (6 vs. 10)	(27.1 ± 3.9 vs. 24.7 ± 4.1)	P3, Pz, P4, O1, O2	Upper α	5 (25)	CST	
Escolano et al., 2012	Two-arm RCT (Control vs. α)	19 (9 vs. 10)	(24.3 ± 3.6 vs. 25.8 ± 4.0)	P3, Pz, P4, O1, O2	Upper α	1 (25)	PASAT	
Escolano et al., 2014	Two-arm RCT (Control vs. α)	19 (9 vs. 10)	(24.3 ± 3.7 vs. 25.8 ± 4.1)	P3, Pz, P4, O1, O2	Upper α	1 (25)	MRT	
Farnia et al., 2017	Three-arm RCT (Control vs. low α/high α)	30 (10 vs. 10)	(34.2 ± 5.7 vs. 31.7 ± 6.4)	FCz	Low α/high α	10 (30)		WMS-R
Gordon et al., 2019	Six-arm RCT (Control vs. α)	165 (40 vs. 20)	(21.9 ± 2.5 vs. 21.6 ± 2.4)	Pz	Upper α	10 (21)	MRT	
Guez et al., 2014	Three-arm RCT (Control vs. α)	30 (10 vs. 10)	23.6 ± 2.7	C4, Pz	Upper α	10 (30)	VMT	Word pair
Hsueh et al., 2016	Two-arm RCT (Control vs. α)	50 (25 vs. 25)	(21.6 ± 2.4 vs. 20.9 ± 2.8)	C3a, C3p, Cza, Czp, C4a, C4p	α	12 (36)	BDST	Word pair
Lecomte and Juhel, 2011	Three-arm RCT (Control vs. α)	30 (10 vs. 10)	75.25	C3, C4, Cz	Upper α	4 (30)		Word pair
Nan et al., 2012	Two-arm RCT (Control vs. α)	32 (16 vs. 16)	23.2 ± 3.1	Cz	α	20 (3.33)	BDST	
Naas et al., 2019	Two-arm RCT (Control vs. α)	33 (16 vs. 17)	21.2 ± 1.4	P7, P8, O1, O2	Upper α	4 (15)	FDST	
Pei et al., 2018	Two-arm trial (Control vs. α)	20 (10 vs. 10)	(21.2 ± 1.7 vs. 22.7 ± 1.9)	Fz, C4	α	5 (36)	BDST	Word pair
Reis et al., 2016	Four-arm RCT (Control vs. α+ θ)	34 (6 vs. 9)	65.9 ± 6.6	FCz, Cz	α + θ	8 (30)	M. Rot.	
Wei et al., 2017	Two-arm RCT (Control vs. α)	30 (15 vs. 15)	26 ± 3	C3	α	12 (25)	BDST	Word pair
Xiong et al., 2014	Four-arm RCT (Control vs. θ/α)	48 (12 vs. 12)	NA (young adult)	Fz, FCz, Cz, C1, C2	θ/α	5 (2)	2-back task	
Zoefel et al., 2011	Two-arm trial (Control vs. α)	24 (10 vs. 12)	(22.1 ± 3.8 vs. 23.7 ± 2.3)	P3, Pz, P4, O1, O2	Upper α	5 (25)	MRT	

*BDST, backward digital span task; CST, conceptual span test; EEG, electroencephalography; EM, episodic memory; FDST, forward digital span task; M. Rot, matrix rotation task; MRT, mental rotation task; NA, not available; NFT, neurofeedback training; PASAT, paced auditory serial addition task; RCT, randomized controlled trials; VMT, verbal memory test; WMS, Wechsler memory scale; WM, working memory*.

The studies varied in the intervention protocols, with differences in feedback modalities, electrode locations, duration of a session, and number of sessions (Table 1). Recorded electrodes were primarily placed over the parietal or fronto-parietal cortices (*n* = 11, 68.75%), and five studies (31.25%) recorded parieto-occipital cortices. Of these studies, nine studies used feedback of upper alpha activity (10–12 Hz), 4 studies used feedback of the full range of alpha (8–12 Hz) activity, 1 study used feedback of low alpha (7–9.5 Hz)/high alpha (9.5–12 Hz) activity, 1 study used feedback of theta/alpha ratio, and 1 study used feedback of alpha and theta activities. The training duration of a session was in the range of 2–36 min and varied among the recruited studies. The number of training sessions was in the range of 1–20 and differed among the recruited studies.

### Risk of Bias Within Studies

**Figure 2:**
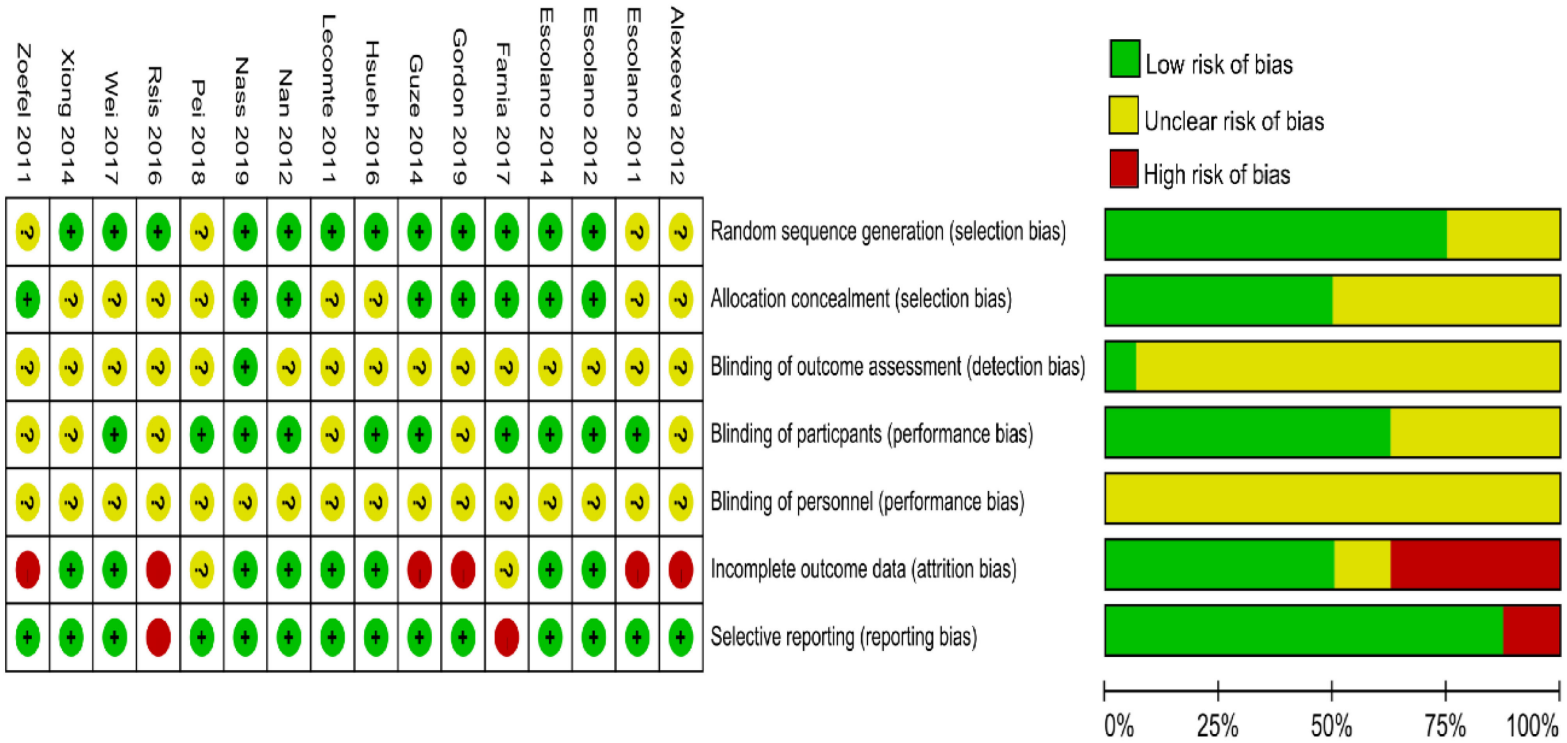
Cochran risk of bias tool. Each risk of bias item for each included study and presented as percentages across all included studies.

Figure 2 shows a summary of the RoB assessment. The level of risk was low for most items in the RoB domains within studies. The majority of studies were categorized as having a low risk of bias in the randomization, allocation, blinding of participants, incomplete outcome data, and selective reporting. An unclear risk of bias was found in the blinding of outcome assessment in 15 of 16 studies (93.75%). Sixteen of 16 studies (100%) exhibited unclear bias in the blinding of personnel.

### Risk of Bias Across Studies

**Figure 3:**
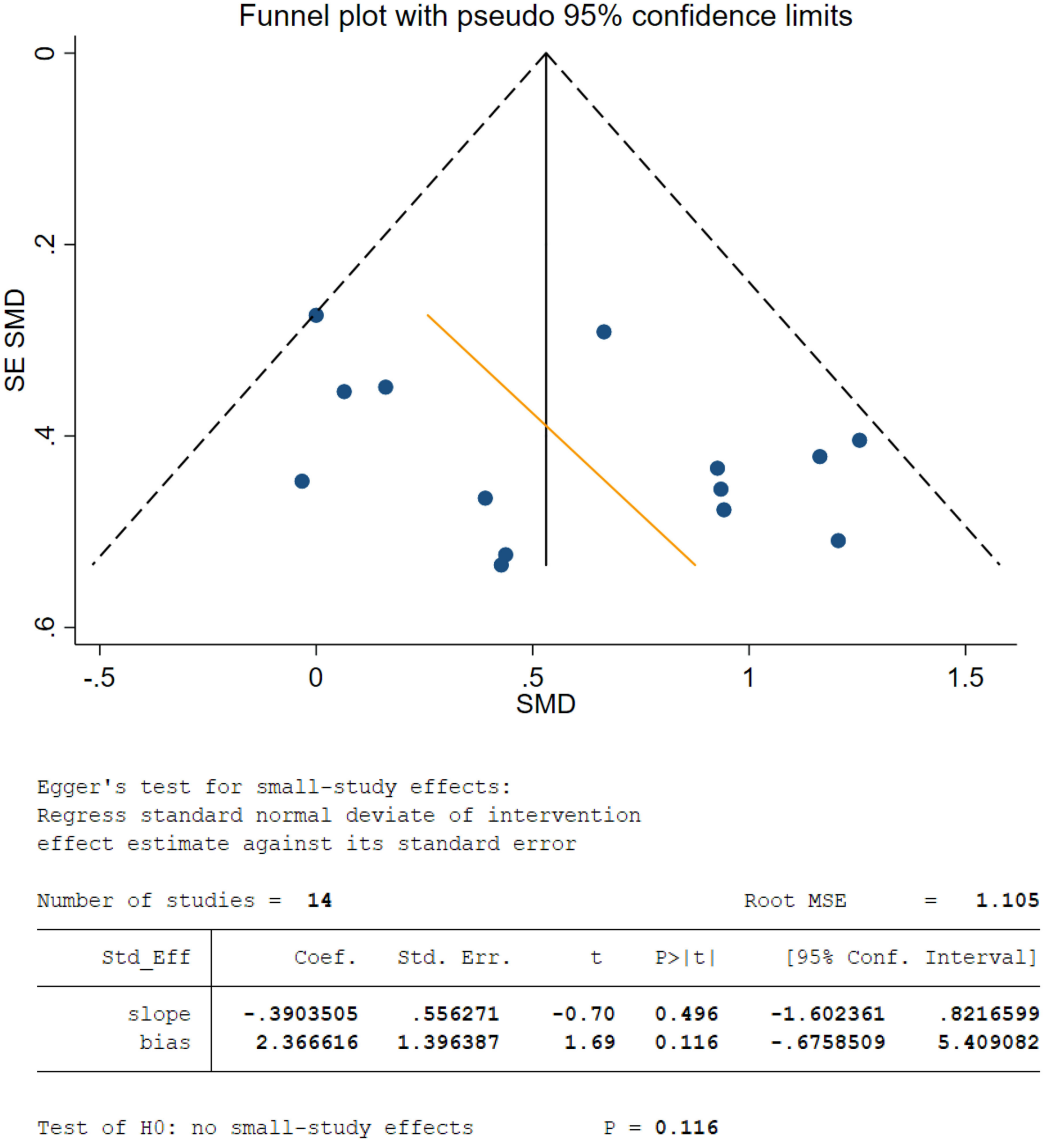
Funnel plot and Egger's test for meta-analysis of effects of alpha neurofeedback training (NFT) on working memory. Each point represents an independent study for the indicated associate. Coef, coefficient; MSE, mean standard error; SE, standard error; SMD, standardized mean difference; Std_eff, standard effects.

The shape of the funnel plot was prone to be symmetrical (Figure 3). There was no significant difference (*P* = 0.116) by the Egger test, suggesting no publication bias among the studies in the WM of healthy participants. On the other hand, only 6 studies of EM were found. Funnel plots are limited for further analysis because of fewer than 10 studies.

### Synthesis of Results

#### Working Memory (WM)

**Figure 4:**
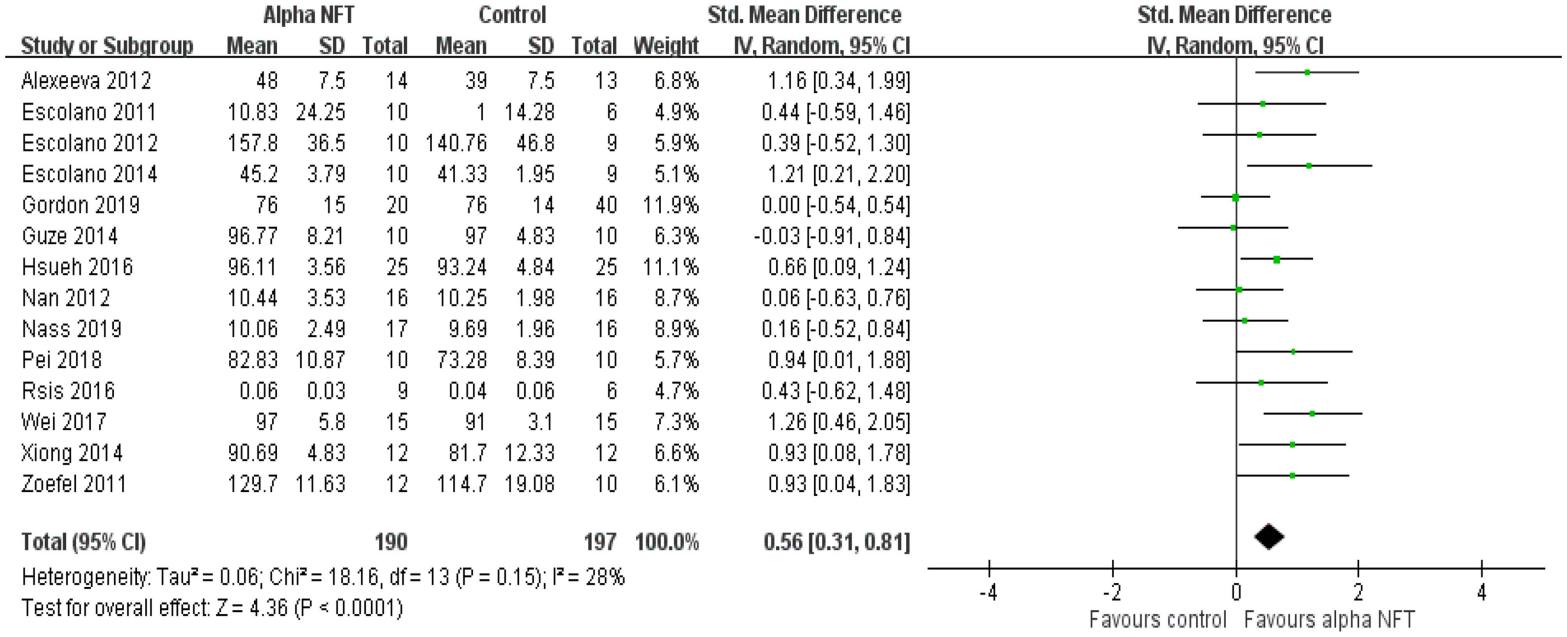
Forest plots for meta-analysis of alpha NFT on working memory in healthy participants. SD, standard deviation; Std, standardized.

Figure 4 shows NFT of alpha activity on the WM of healthy participants in fourteen of 16 studies. Of these WM studies, 8 of 14 studies (57.1%) exhibited significant WM enhancement compared with the control group. The results showed a significant overall effect with an SMD of 0.56 (95% CI 0.31–0.81, *P* < 0.0001, *I*^2^ = 28%), suggesting that NFT of alpha activity would improve WM performance compared with the control group.

Regarding sample size, seven of 14 studies (50%) recruited ≤ 10 participants (9–10) into the alpha group and ≤ 10 participants (6–10) into the control group. The results showed a significant overall effect with an SMD of 0.60 (95% CI 0.25–0.96, *P* = 0.001, *I*^2^ = 0%). Seven of 14 studies (50%) recruited >10 participants (12–25) into the alpha group. The results exhibited a significant overall effect with an SMD of 0.55 (95% CI 0.17–0.94, *P* = 0.005, *I*^2^ = 54%).

With regard to electrode placement, nine of 14 studies (64.3%) placed electrodes over the parietal or fronto-parietal cortices. The results showed a significant overall effect with an SMD of 0.57 (95% CI 0.23–0.91, *P* = 0.001, *I*^2^ = 44%). Five of 14 studies (35.7%) placed electrodes over the parieto-occipital cortices. The results showed a significant overall effect with an SMD of 0.55 (95% CI 0.16–0.94, *P* = 0.006, *I*^2^ = 0%).

Regarding the type of brain activity used to compute the feedback, four of 14 studies (28.6%) used an entire alpha amplitude. The results showed a significant overall effect with an SMD of 0.69 (95% CI 0.20–1.18, *P* = 0.005, *I*^2^ = 44%). Eight of 14 studies (57.1%) selected feedback of an upper alpha amplitude and showed a significant overall effect with an SMD of 0.47 (95% CI 0.11–0.82, *P* = 0.01, *I*^2^ = 35%). One of 14 studies (7.1%) evaluated a theta/alpha value, and one of 14 studies (7.1%) investigated feedback of alpha and theta activities. The two papers exhibited significant WM improvement.

Regarding the duration of a session, four of 14 studies (28.6%) designed a session of ≤ 20 min (2–18 min). The results showed a significant overall effect with an SMD of 0.53 (95% CI 0.00–1.07, *P* = 0.05, *I*^2^ = 49%). Ten of 14 studies (71.4%) conducted a session of >20 min. The results showed a significant overall effect with an SMD of 0.58 (95% CI 0.28–0.88, *P* = 0.0002, *I*^2^ = 26%).

We further considered the influence of the amount of sessions. Two of 14 studies (14.3%) used a single-session NFT. The results showed a significant overall effect with an SMD of 0.77 (95% CI −0.03–1.57, *P* = 0.05, *I*^2^ = 28%). Twelve of 14 studies (85.7%) performed alpha NFT with 4–20 sessions and showed a significant overall effect with an SMD of 0.54 (95% CI 0.26–0.81, *P* = 0.0001, *I*^2^ = 32%).

Finally, we considered the age effect of alpha NFT on WM. Thirteen of 14 studies (92.9%) recruited young adults. The results showed a significant overall effect with an SMD of 0.57 (95% CI 0.30–0.84, *P* < 0.0001, *I*^2^ = 34%). Only one study (7.1%) recruited elderly individuals to test alpha NFT on WM. There was no significant difference between the alpha group and the control group.

#### Episodic Memory (EM)

**Figure 5:**
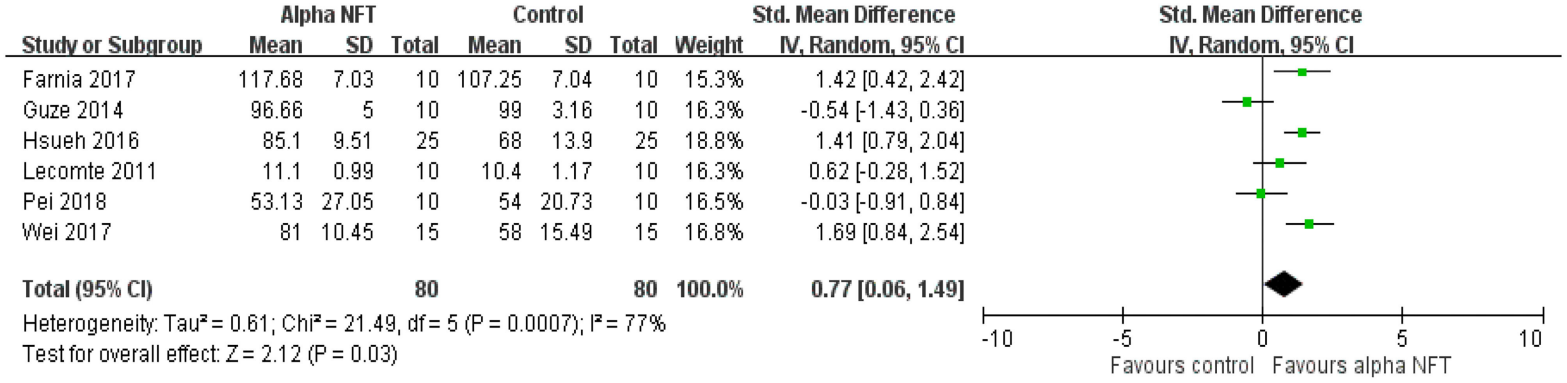
Forest plots for meta-analysis of alpha NFT on episodic memory in healthy participants. SD, standard deviation; Std, standardized.

Figure 5 shows NFT of alpha activity on the EM of healthy participants in six of 16 studies. Of these EM studies, 2 of 6 studies (33%) exhibited significant EM enhancement compared with the control group. The results showed a significant overall effect with an SMD of 0.77 (95% CI 0.06–1.49, *P* = 0.03, *I*^2^ = 77%), suggesting that NFT of alpha activity could improve EM performance compared with the control group.

Six studies placed recording electrodes over the parietal or fronto-parietal cortices. All studies used a session of ≥20 min (25–36 min). Five of 6 studies (83.3%) recruited young adults. The results showed a marginally significant overall effect with an SMD of 0.8 (95% CI −0.06–1.66, *P* = 0.07, *I*^2^ = 81%). Only one study (16.7%) recruited elderly individuals to test alpha NFT on EM. There was no significant difference between the alpha group and the control group.

Regarding the sample size, four of 6 studies (66.7%) recruited ≤ 10 participants into the alpha group. The results showed no overall effect with an SMD of 0.35 (95% CI −0.46–1.15, *P* = 0.4, *I*^2^ = 67%). Two of 6 studies (33.3%) recruited >10 participants (15–25) into the alpha group. The results showed a significant overall effect with an SMD of 1.51 (95% CI 1.01–2.02, *P* < 0.00001, *I*^2^ = 0%).

Three of 6 studies (50.0%) used feedback of an entire alpha amplitude. The results showed a significant overall effect with an SMD of 1.05 (95% CI 0.08–2.01, *P* = 0.03, *I*^2^ = 78%). Two of 6 studies (33.3%) selected feedback of an upper alpha amplitude, and showed no overall effect with an SMD of 0.04 (95% CI −1.09–1.17, *P* = 0.94, *I*^2^ = 68%). A study (16.7%) investigated feedback of a ratio of low alpha-to-high alpha amplitude and exhibited significant EM improvement.

We further considered the influence of session amount. No study used a single session. Four of 6 studies (66.7%) used ≤ 10 sessions. The results showed an insignificant overall effect with an SMD of 0.35 (95% CI −0.46–1.15, *P* = 0.4, *I*^2^ = 67%). Two of 6 studies (33.3%) used >10 sessions. The results showed a significant overall effect with an SMD of 1.51 (95% CI 1.01–2.02, *P* < 0.00001, *I*^2^ = 0%).

## Discussion

Sixteen clinical trials were included in this study involving 427 participants (217 control vs. 210 alpha NFT). We found the following: (1) RoB assessment in random sequence generation, allocation concealment, blinding of participants, incomplete outcome data, and selective reporting was categorized as low risk of bias. Blinding of outcome assessment and personnel were mostly categorized as unclear risk of bias; (2) studies of alpha NFT on WM exhibited no significant publication bias; (3) Alpha NFT remarkably improved the WM of healthy participants; (4) Alpha NFT significantly improved EM in healthy participants. These results point to a positive effect of alpha NFT on memory performance in healthy participants.

In general, the small sample size and heterogeneity in the treatment protocols of the included studies are believed to affect outcomes. The meta-analysis results indicated that a large sample size (>10) produced a significant overall effect of alpha NFT on WM and EM. A small sample size (≤ 10) in the alpha group resulted in a significant overall effect in WM but insignificance in EM. The results may be due to the different effect sizes of alpha NFT and heterogeneous cognitive tasks of WM and EM. These phenomena reflect the varied effectiveness of NFT on memory in small sample sizes. Our results suggest that the sample size of a group should be more than 10 healthy participants for alpha NFT on memory.

In addition, there was considerable variability in the intensity and dose of NFT among studies. Most NFT studies conducted sessions of ~30 min, particularly for EM studies. For WM studies, studies with session duration of ≤ 20 min attained a marginally significant level. Alpha NFT with a duration of >20 min *per session* exhibited a significant overall effect in both WM and EM. The results indicate that a suitable duration for a session for alpha NFT on memory may be longer than 20 min.

Alpha NFT with a single session or more sessions exhibited a significant effect on WM. The results suggest an immediate advantage of alpha NFT on the WM process. Previous studies have indicated enhanced alpha activity during retention of WM, which is supported by a positive correlation of alpha amplitude with the WM load (Jensen et al., 2002) and the difficulty of the WM task (Sauseng et al., 2005). Similarly, the sensorimotor rhythm of a single-session NFT facilitates early acquisition of a procedural motor task (Ros et al., 2014). The alpha amplitude can reflect an optimal filter to detect weak incoming stimuli in a psychophysical task (Linkenkaer-Hansen et al., 2004) and may improve the WM process.

Regarding EM, there was an insignificant overall effect for alpha NFT with ≤ 10 sessions. Alpha NFT of >10 sessions exhibited a significant effect on EM. These results suggest that numerous NFT sessions, such as >10 sessions, are required for EM enhancement.

Alpha NFT of young adults exhibited a significant effect on WM or marginal significance on EM. Two studies using elderly participants exhibited no significant improvement in EM using NFT of 4 sessions (Lecomte and Juhel, 2011) or WM using NFT of 8 sessions (Reis et al., 2016). Aging is associated with decreased alpha frequency and diminished alpha amplitude (Duffy et al., 1984). This finding partially reflects the importance of increasing alpha activity-related variables for NFT in elderly populations. Alpha NFT in older people with dementia exhibits a considerable improvement of learning and past memory with 30 sessions of training (Luijmes et al., 2016). This phenomenon implies that aging participants need more NFT sessions to produce effectiveness on memory.

Our results indicate that alpha NFT exhibited a significant effect on WM regarding to feedback of alpha activity-related indexes in previous studies. The phenomenon may support a positive association between alpha activity and intelligence (particular for WM item) (Doppelmayr et al., 2002). On the other hand, feedback of an entire alpha amplitude exhibited a significant effect on EM, but feedback of an upper alpha amplitude showed no effect. Meanwhile, feedback of a ratio of low alpha-to-high alpha amplitude presented a significant effect on EM. These results propose a possibility for NFT of the low alpha activity on EM enhancement. Low alpha activity has a higher positive association with performance of word pair task compared with that of the upper alpha activity in the LGT-3 intelligence measure (Doppelmayr et al., 2002). Moreover, inhibition or desynchronization of the upper alpha activity reflects a better performance of semantic memory (Klimesch et al., 1999). Taken together, NFT of alpha activity, particular for the low alpha range, may play an important role in EM enhancement.

Recently, the NFT effect has raised an issue about real treatment effects or placebo results (Schabus et al., 2017; Pigott et al., 2018). In our meta-analyses, the included studies of alpha NFT were restricted to a two-group randomized experimental design with a control group. WM and EM exhibited significant enhancement in 8 of 14 studies (57.1%) and 2 of 6 studies (33%) compared with the control group, respectively. The meta-analysis results suggest a significant increase in accuracy in both WM and EM of the alpha group compared with that of the control group. The results indicate a positive effect of the alpha NFT on memory. In addition, the control groups of most included studies (9 of 14 WM studies and 4 of 6 EM studies) (1 study with active neurofeedback (Pei et al., 2018), 2 studies with sham neurofeedback (Guez et al., 2014; Xiong et al., 2014), or 8 studies with silent feedback (Escolano et al., 2011; Lecomte and Juhel, 2011; Zoefel et al., 2011; Alekseeva et al., 2012; Reis et al., 2016; Farnia et al., 2017; Gordon et al., 2019; Naas et al., 2019) showed no memory change. Two active control groups showed significant improvement in WM and EM after training (Hsueh et al., 2016; Wei et al., 2017), and the alpha group of the two studies exhibited significant enhancement of WM and EM compared with the active control group. The results may suggest little placebo influence of an NFT route. Overall, alpha NFT produced a realistic contribution to memory enhancement in our meta-analyses.

A meta-analysis provides constructive information and conclusive remarks for specific issues. The current study found advantages of alpha NFT on both WM and EM. A more confident interpretation for the meta result can be found with >10 included studies (van Wely, 2014). In the present study, only 6 EM studies with alpha NFT met criteria. Although several parameters, such as >10 training sessions, >10 sample size, >20 min duration *per session*, and feedback of an entire alpha amplitude, play an important role in EM enhancement by alpha NFT. More studies are required to increase interpretation power for effect of alpha NFT on EM.

The present study exclusively searched alpha NFT studies with healthy participants. Our meta-analysis results suggest a conclusive positive effect of alpha NFT on memory. Previous meta-studies have indicated largely controversial observations about NFT on cognitive function (including memory) in different populations, such as patients with stroke (Renton et al., 2017), posttraumatic stress disorder (Cramer et al., 2018; Steingrimsson et al., 2020), and attention-deficit/hyperactivity disorder (Cortese et al., 2016). Heterogeneity exists in the recruited population, NFT protocols, measuring outcomes, training paradigms, and experimental design. These factors raise the difficulty of interpreting the NFT effect. In general, neurological or psychiatric disorders cause substantial changes in the brain network, which may resist NFT progression and limit the improvement of cognitive ability. Caution is needed when generalizing the findings of this paper.

Our meta results indicate alpha NFT on memory enhancement, but memory improvement does not exhibit in all previous studies. Studies are lacking in analyzing memory performance between successful and non-successful training participants. Participants with successful training to controlling alpha activity (or called “Responder”) demonstrated better WM and EM performance compared with those of entire alpha group (Hsueh et al., 2016). It may indicate less successful alpha training leading to increased variance of memory measures.

Another controversial result is baseline alpha activity for an NFT since most studies lack measure of baseline alpha activity. Some studies provide evidence of increased baseline alpha activity throughout the training to demonstrate a successful NFT (Escolano et al., 2011; Zoefel et al., 2011). Spontaneous alpha activity is related to memory performance (Doppelmayr et al., 2002), and resting alpha activity can predict a learning ability of an NFT (Wan et al., 2014). They may echo the relationship between baseline alpha activity and successful NFT. On the other hand, some studies present no change in baseline alpha activity throughout the training (Nan et al., 2012; Hsueh et al., 2016; Wei et al., 2017). Of these studies the baseline alpha activity is considered as a quality control of EEG, and participants are recorded under a resting condition without neurofeedback scenario. They have shown progressive increase in alpha amplitude throughout the training as a success index. These studies emphasize a great controllability of alpha activity instead of baseline alpha alteration for NFT (Hsueh et al., 2016).

Possible adverse reactions, such as fatigue, sleep disturbance, seizure, anxiety, or depression, from NFT of different brain rhythms exhibit in a few patients (Hammond and Kirk, 2008). Of studies with alpha NFT in healthy participants, most had no specific comment on transient side effect or serious adverse reaction. A study of alpha NFT (Hsueh et al., 2016) evaluated anxiety, depression, and insomnia with adequate questionnaires and showed no deterioration of these psychiatric symptoms after NFT. It remains to be investigated whether other aspects of adverse effects exist due to alpha NFT.

## Caution for Alpha on Memory

Caution is required when interpreting these findings given a number of limitations in addition to the issues raised with regard to the nature of the trials. First, effect size estimates may be inflated because of the failure to report incomplete outcome data. Second, there were insufficient trials measuring important outcomes, such as intelligence quotient and academic skills. Third, there were incomplete demonstrations of important indexes, e.g., baseline alpha activity and spectral characteristics throughout the training, in most trials. Finally, the level of methodological rigor specifically related to RCT conduct by the RoB tool was generally unclear. The level of blinding was insufficient in many studies. A complementary checklist for neurofeedback trials, including guidelines of pre-experiment, control groups and measures, feedback specifications, and outcome measures, will be important to improve level of evidence of alpha NFT (Ros et al., 2020).

## Conclusion

We explored and analyzed all randomized controlled trials to provide a complete and helpful overview of alpha NFT intervention on memory in healthy participants. Our data show a positive contribution of alpha NFT on memory, including WM and EM. Alpha NFT could be added as a potential selection in current memory trainings. It may also contribute to the enhancement of several aspects of cognitive functions. The impacts of alpha NFT on cognition and neurological/psychiatric symptoms need further larger, adequately powered studies.

## Data Availability Statement

The original contributions presented in the study are included in the article/supplementary materials, further inquiries can be directed to the corresponding author/s.

## Author Contributions

W-HY and F-ZS were involved in project administration, study conceptualization, data curation, and data interpretation. W-HY wrote the first draft of the manuscript and conducted data collection and analysis. J-JH screened the literature. All authors were involved in the supply of the materials, conducted critical revision, and approved the final version for publication.

## Conflict of Interest

The authors declare that the research was conducted in the absence of any commercial or financial relationships that could be construed as a potential conflict of interest.
